# Prize Day at the London

**Published:** 1889-07-20

**Authors:** 


### Prize Day at the London.

There was high festival at the London Hospital on Monday, the 15th. The Duke of Cambridge, president of the hospital, came to present Sir Andrew Clark with his portrait, painted by the late Frank Holl, R.A., and subscribed for by the medical and surgical staff of the hospital. He then presented the Hospital Medical College with a replica of the portrait, painted by Mr. Sidney Hodges, who undertook the task when Mr. Holl died without fulfilling this part of his commission, and who has produced an admirable copy. This being done, and the gifts duly acknowledged by Sir Andrew Clark and Mr. J. H. Buxton, His Royal Highness presented the prizes to the students, and then to the probationer nurses who had distinguished themselves in the recent examinations. The students and nurses filled one side of the library, where the ceremony took place, and on the platform were Mr. Carr-Gomm, Mr. J. H. Buxton, Sir John Watson, Mr. Munro Scott, Mr. Jonathan Hutchinson, who was enthusiastically received on this his first public appearance since his election to the presidency of the Royal College of Surgeons, and the rest of the staff of the hospital. Miss Lückes sat with the visitors in the body of the hall, and we were glad to notice that Sir Andrew Clark referred to her able and kindly management of the nursing department in high terms of commendation, whilst bearing testimony to its efficiency and remarkable success.

The Duke of Cambridge having made the presentation in a few appropriate words,

Sir Andrew Clark replied in an interesting and excellent speech. He traced the development of the medical school of the hospital from its first beginning in 1742, when the medical staff first conceived the idea of imparting the knowledge they had gained. In 1749 lectures were begun, and soon after the staff clubbed together and built, at their own expense, and on their own responsibility, a little school at the east end of the hospital. There Sir Andrew Clark began that thirty-five years' service of the London Hospital which ended on Monday, when he retired from the active staff to assume the position of consulting physician. In 1834, however, the school had developed so much that the "little school," enlarged as much as possible, was insufficient, and the governors erected the original London Hospital Medical College, and handed it over to the medical staff. In 1885 a new building was again required, which was opened by the Prince of Wales in 1887. From its early days the London Hospital was noted for the practical nature of its teaching. That was the characteristic by which it was known in the classrooms of Edinburgh and Paris. It was the first school in England where medicine was taught as a handicraft, and not merely from books. Here were delivered the first clinical lectures in England, and Sir Andrew first taught the use of the microscope in the diagnosis of disease, and established the first physiological laboratory in England. The result of this method of teaching may be guessed from the fact that in the Crimean War the men who went from the London Hospital proved trustworthy, practical surgeons, and at the present moment the presidents of the Royal Colleges of Physicians and Surgeons are both teachers at the London. Going into personal reminiscences, Sir Andrew spoke of his coming to London in 1853, very young, almost friendless, and in bad health. When he received the appointment of assistant physician to the London Hospital, the friends of his defeated opponent said, "Poor Scotch beggar, let him have it; he can't possibly live six months!" But Sir Andrew Clark having lived rather more than six months, attributes his success to his Scotch self-dependence, and his earnest love of work for work's sake.

The probationers who received prizes were Misses Bishop, Ridal, and Wheeler, while certificates were granted to Misses Collinson, Herrman, Huntley, and Yatman, who had come within one mark of the last prize-taker.

